# Physiological and Pharmaceutical Considerations for Rectal Drug Formulations

**DOI:** 10.3389/fphar.2019.01196

**Published:** 2019-10-16

**Authors:** Susan Hua

**Affiliations:** ^1^Therapeutic Targeting Research Group, School of Biomedical Sciences and Pharmacy, University of Newcastle, Callaghan, NSW, Australia; ^2^Hunter Medical Research Institute, New Lambton Heights, NSW, Australia

**Keywords:** rectal, rectum, drug delivery, dosage form, nanoparticles, drug formulation, physiological considerations, translation

## Abstract

Although the oral route is the most convenient route for drug administration, there are a number of circumstances where this is not possible from either a clinical or pharmaceutical perspective. In these cases, the rectal route may represent a practical alternative and can be used to administer drugs for both local and systemic actions. The environment in the rectum is considered relatively constant and stable and has low enzymatic activity in comparison to other sections of the gastrointestinal tract. In addition, drugs can partially bypass the liver following systemic absorption, which reduces the hepatic first-pass effect. Therefore, rectal drug delivery can provide significant local and systemic levels for various drugs, despite the relatively small surface area of the rectal mucosa. Further development and optimization of rectal drug formulations have led to improvements in drug bioavailability, formulation retention, and drug release kinetics. However, despite the pharmaceutical advances in rectal drug delivery, very few of them have translated to the clinical phase. This review will address the physiological and pharmaceutical considerations influencing rectal drug delivery as well as the conventional and novel drug delivery approaches. The translational challenges and development aspects of novel formulations will also be discussed.

## Introduction

The oral route is the most convenient route for drug administration. However, there are circumstances where this is not possible from either a clinical or pharmaceutical perspective (de Boer et al., 1982; De Boer et al., 1984). In these cases, the rectal route may represent a practical alternative and can be used to administer drugs for both local and systemic actions. The rectal route is already used clinically to deliver a variety of therapies to treat both local (**Table 1**) and systemic conditions (**Table 2**). This includes the local treatment of constipation, hemorrhoids, anal fissures, inflammation, and hyperkalemia. Rectal formulations for systemic drug delivery are used clinically for the treatment of pain, fever, nausea and vomiting, migraines, allergies, and sedation. These rectal formulations are based on conventional dosage forms, such as suppositories and enemas, and are typically used for short-term therapy.

**Table 1:** Examples of rectal formulations clinically approved for local absorption.

Drug	Brand name	Indication	Dosage form
Bisacodyl	*Dulcolax* *Bisalax*	Constipation	Suppository Enema
Glycerol	*Glycerol*	Constipation	Suppository
Saline laxatives	*Micolette* *Microlax*	Constipation Bowel preparation	Enema
Mesalazine	*Pentasa* *Salofalk*	Inflammatory bowel disease	Suppository Enema Rectal foam
Budesonide	*Budenofalk*	Anti-inflammatory	Rectal foam
Prednisolone	*Colifoam*	Anti-inflammatory	Rectal foam
Hydrocortisone	*Predsol* *Colocort*	Anti-inflammatory	Suppository Enema
Polystyrene sulfonate resins	*Resonium A*	Hyperkalemia	Enema
Glyceryl trinitrate	*Rectogesic*	Anal fissure, hemorrhoids	Ointment

**Table 2:** Examples of rectal formulations clinically approved for systemic absorption.

Drug	Brand name	Indication	Dosage form
Acetaminophen	*Panadol* *Acephen* *Feverall*	Pain, fever	Suppository
Oxycodone	*Proladone*	Pain	Suppository
Ondansetron	*Zofran*	Nausea and vomiting	Suppository
Caffeine + ergotamine	*Migergot*	Migraine	Suppository
Prochlorperazine	*Compro*	Nausea and vomiting	Suppository
Promethazine	*Phenergan*	Antihistamine	Suppository
Ibuprofen	*Nurofen*	Pain, fever	Suppository
Diclofenac	*Voltaren*	Pain, fever	Suppository
Indomethacin	*Indocin*	Pain	Suppository
Diazepam	*Diazepam rectal solution* *Diastat AcuDial*	Seizures, sedation	Enema Gel

Rectal dosage forms are generally inexpensive to manufacture and can also be self-administered by patients without the need for a medically trained person in comparison to parenteral dosage forms (e.g., intramuscular and intravenous injections) (Turner et al., 2012; Jannin et al., 2014). This is particularly advantageous for rural communities and developing countries for specific drugs that cannot be delivered by other convenient routes (Abolhassani et al., 2000; Turner et al., 2012; Jannin et al., 2014). However, the rectal route of administration is generally not preferred by patients due to cultural issues and/or potential for discomfort and leakage (de Boer et al., 1982; De Boer et al., 1984; Jannin et al., 2014; Nunes et al., 2014). These factors have contributed to (i) a lack of drugs that are clinically available in rectal dosage forms, (ii) a lack of clinical conditions that are treated with rectal drug formulation, and (iii) a lack of comprehensive bioavailability studies in humans (Jannin et al., 2014).

Further development and optimization of rectal drug formulations have led to improvements in drug availability (i.e., locally and systemically), formulation retention, and drug release kinetics (e.g., rapid or controlled release). However, despite the pharmaceutical advances in rectal drug delivery, very few of them have translated to the clinical phase. This review will address the physiological and pharmaceutical considerations influencing rectal drug delivery as well as the conventional and novel drug delivery approaches. The translational challenges and development aspects of novel formulations will also be discussed.

## Functional Anatomy

The rectum is the final portion of the large intestine that starts from the end of the sigmoid colon to the anal canal. It primarily acts as a transportation (conduit) or temporary storage site in the defecation process, with only minimal involvement in the absorption of water and electrolytes from the gastrointestinal contents (Shafik et al., 2006; Leppik and Patel, 2015). Fecal matter is stored by the rectum if it is small in volume until it reaches a degree of rectal distension sufficient to initiate the defecation reflex (Shafik et al., 2006). The main anatomical difference between the rectum of adults and children is based on size. The length of the rectum is ∼15–20 cm in adults, with a surface area of around 200–400 cm^2^ (de Boer et al., 1982; van Hoogdalem et al., 1991; Nunes et al., 2014). In children, further size differences are evident due to the developing gastrointestinal tract. For example, the rectum is ∼3 cm in length and has a surface area of ∼18 cm^2^ at 1 month of age compared to ∼12 cm in length and ∼230 cm^2^ at 10 years of age (Woody et al., 1989; Jannin et al., 2014). Although the rectum is formed at birth, it is only functional when the baby starts to feed orally (Jannin et al., 2014).

In general, the environment in the rectum is relatively constant and static in comparison to other parts of the gastrointestinal tract (Jannin et al., 2014). The rectum has an average fluid volume of 1–3 ml and a neutral pH of 7–8, with minimal buffering capacity (Evans et al., 1988; Jannin et al., 2014; Nunes et al., 2014; Purohit et al., 2018). There have been conflicting reports regarding the rectal pH in children. Jantzen et al. (1989) measured the rectal pH in 100 healthy pediatric patients (25 infants and 75 children). Mean rectal pH was reported as 9.6; however, there was a wide range in rectal pH values (pH 7.2–12.1). Conversely,Turner et al. (2012) reported an average rectal pH of 6.75 from 100 well and 45 unwell infants, with no significant difference between the two groups. Interestingly, the mean rectal pH of well neonates (pH 6.47) was significantly lower than that of older infants (> 28 days of age, pH 6.90). The reason for the discrepancy in results may be due to physiological or technical differences in the studies; however, they should still be considered when developing or evaluating rectal dosage forms for pediatric patients, as this may affect drug partitioning and absorption. In addition, although the colon contains the majority of the gastrointestinal microbiome, it has been suggested that some residual bacterial enzymes are found in the rectum (Sartor, 2008; Macfarlane and Macfarlane, 2011). However, presystemic loss of drug by intraluminal degradation by microorganisms or metabolism within the mucosal cells in the rectum is generally not considered to be significant (de Boer et al., 1982; De Boer et al., 1984; Jannin et al., 2014).

In terms of histology, the rectal mucosa forms the innermost layer of the rectum that is in contact with fecal matter. The rectum does not have villi or microvilli on the luminal surface, hence the relatively small surface area for absorption in comparison to the small intestine (van Hoogdalem et al., 1991; Nunes et al., 2014; Reinus and Simon, 2014). The mucosal surface of the rectum is structured with a single layer of columnar cells to form the epithelium (Reinus and Simon, 2014). The epithelium also consists of numerous goblet cells that are interspersed among the absorptive cells (Reinus and Simon, 2014). Goblet cells are important for secreting mucus, which protects the rectal epithelia and helps to lubricate fecal matter as they pass through the rectum. At the anorectal junction, the mucosa transitions to non-keratinized stratified squamous epithelium and eventually to keratinized stratified squamous epithelium at the external anal sphincter (Nunes et al., 2014; Reinus and Simon, 2014).

The rectal region is drained by rectal (hemorrhoidal) veins and lymphatic vessels (de Boer et al., 1982; De Boer et al., 1984; van Hoogdalem et al., 1991; Dujovny et al., 2004; Nunes et al., 2014; Purohit et al., 2018). The superior rectal vein drains the upper part of the rectum, and the inferior and middle rectal veins drain the lower part of the rectum. More specifically, the superior rectal vein drains into the portal vein, which passes the blood through the liver prior to reaching the systemic circulation. In contrast, the inferior and middle rectal veins drain into the inferior vena cava and, therefore, directly into the systemic circulation. Between these three rectal veins exist extensive anastomoses, which connect all three veins throughout the rectum. The rectum is also extensively drained by the lymphatic system that originates in the mucosa and submucosa. The influence of the lymphatic vessels on the absorption of drugs is not well established; however, it may contribute to the systemic absorption of highly lipophilic drugs (Jannin et al., 2014; Nunes et al., 2014; Purohit et al., 2018). Lymphatic drainage also avoids the hepatic first-pass effect (de Boer et al., 1982; van Hoogdalem et al., 1991).

## Comparison of the Rectal Route of Administration to Other Sections of the Gastrointestinal Tract

For a balanced view of rectal drug delivery in the clinical setting, it is important to compare this route of drug administration to other sections of the gastrointestinal tract. In general, the oral route is the most preferred by patients, due to its advantages such as ease of use, non-invasiveness, and convenience for self-administration (Shreya et al., 2018; Homayun et al., 2019). The major site of drug absorption following oral administration is the small intestine, which has a much larger surface area compared to the rectum (Marieb and Hoehn, 2010; Reinus and Simon, 2014). Although the small intestine has been estimated to have a surface area of 200 m^2^ in an adult, recent reports have suggested this to be more closer to approximately 32 m^2^ for the interior of the gastrointestinal tract, with approximately 2 m^2^ representing the large intestine (Helander and Fandriks, 2014). However, drugs administered orally can be unpleasant in taste, cause gastric irritation, and suffer from high first-pass drug elimination processes in the intestine and/or liver (Martinez and Amidon, 2002; Homayun et al., 2019). The physiological environment in the gastrointestinal tract can also affect the stability, solubility, and permeability of drugs, including the acidic gastric pH, gastrointestinal transit time, gastrointestinal mucus, and metabolism through enzymatic or microbial degradation (Martinez and Amidon, 2002; Shreya et al., 2018; Homayun et al., 2019). In addition, oral drug delivery can be challenging as the physiology of the human gastrointestinal tract can display both intra-individual and inter-individual variability (Martinez and Amidon, 2002). Therefore, the oral route of administration is less attractive for drugs that are significantly affected by these conditions.

The rectal route for drug delivery can be useful for drugs that have poor stability, solubility, or permeability following oral administration. It can also be used when oral ingestion is precluded—for example, in patients experiencing nausea and vomiting, when the patient is unconscious, or for patients that have swallowing difficulties (e.g., pediatric and geriatric patients). Although the surface area of the rectum is considerably smaller than that of the small intestine, the environment in the empty rectum is considered relatively constant and stable (Jannin et al., 2014). This favors a reproducible absorption process and has low enzymatic activity as compared to other sections of the gastrointestinal tract. In addition, drugs can partially bypass the liver following systemic absorption, which reduces the hepatic first-pass effect. Therefore, rectal drug formulations can be useful for drugs that: (i) undergo high hepatic first-pass metabolism, (ii) have limited absorption in the upper gastrointestinal tract, (iii) are readily degradable or unstable in the gastrointestinal tract, (iv) cause irritation to the gastric mucosa, (v) cannot be easily formulated for other routes of administration, and (vi) have localized actions in the rectum or distal colon (de Boer et al., 1982; De Boer et al., 1984; Jannin et al., 2014; Nunes et al., 2014).

## Biopharmaceutical Considerations Influencing Rectal Drug Absorption

Drug absorption following rectal administration is determined by a combination of formulation-related factors, drug-related factors, and physiology-related factors. The latter has been covered in other sections of the review (refer to *“Functional anatomy”* and *“Physiological factors influencing rectal drug delivery”*). For absorption to occur, drugs must first be released from the formulation and then be solubilized in the low volume of rectal fluid before crossing the mucus layer and epithelium (van Hoogdalem et al., 1991). This is highly dependent on the formulation, with liquid dosage forms that contain drugs in solution (e.g., enemas) having faster absorption rates in comparison to solid dosage forms (e.g., suppositories and tablets) that require disintegration, liquefaction, and/or dissolution of the formulation to release the drug. Suspended drug particles will then need to dissolve in the luminal fluid before absorption can occur. It should be noted that the drug release rate from the formulation will depend on the partition coefficient of the drug between the vehicle and the aqueous rectal fluid (Nunes et al., 2014). For example, drugs with a high partition coefficient will be more lipophilic. This may lead to slow release of the drug from formulations that have fatty bases in comparison to more hydrophilic bases, which may produce a more sustained release effect. Therefore, the rate limiting step for drug absorption differs based on the formulation and the physical state of the drug in the formulation.

The physicochemical characteristics of the drug will also affect its ability to be absorbed *via* the rectal route. This includes solubility, degree of ionization, partition coefficient, and particle size. Following release from the formulation, the solubility of the drug in the rectal fluid will determine the maximum concentration available for absorption and will also establish a concentration-dependent gradient to drive absorption. In general, higher drug solubility is associated with faster dissolution rates and more rapid absorption. Drug molecules are predominantly transported passively *via* paracellular diffusion (between cells) or transcellular diffusion (through the cell), depending on its physicochemical characteristics. Paracellular transport is preferred for more hydrophilic molecules, ionized molecules, and high molecular weight compounds (Muranishi, 1984; Hayashi et al., 1997; Nunes et al., 2014); however, it can be restricted by the narrow tight junction space (Madara, 1998; Reinus and Simon, 2014). Therefore, the transcellular route is the main mechanism for drug absorption in the rectum (Muranishi, 1984; Hayashi et al., 1997; Nunes et al., 2014). Transcellular diffusion is affected by many factors, but it is usually proportional to the lipid solubility of the drug. Drug molecules in the non-ionized form are much more lipophilic than the ionized form (Allen et al., 2011; Jannin et al., 2014; Nunes et al., 2014; Purohit et al., 2018). At the relatively neutral pH of the rectum, basic drugs with an acid dissociation constant (pKa) near or above the physiologic range tend to be more readily absorbed, as they will predominantly be in their non-ionized form.

Having the optimal balance between hydrophilicity and lipophilicity is important for effective rectal drug delivery. Ideally, drugs should have adequate hydrophilic properties to be soluble in the rectal fluid and be lipophilic enough to cross the epithelium. As many drugs, including more than 40% of new chemical entities, have significant solubility issues in water, various techniques have been investigated to enhance their solubility. This includes particle size reduction, salt formation, use of surfactants, and encapsulation into nanoparticulate formulations (Savjani et al., 2012). In particular, the particle size distribution of active ingredients and excipients is an important physical characteristic of a formulation that has a strong impact on the rate of drug dissolution and absorption (Savjani et al., 2012; Sandri et al., 2014). Smaller particles tend to have higher dissolution rates due to the larger surface area to volume ratio and, therefore, a better chance for faster absorption. The larger surface area allows greater interaction with the solvent, thereby increasing its solubility. It should be noted that particle size has little effect on drugs that are readily water-soluble. However, particles in the size range of 50–100 µm are considered ideal, as they minimize both agglomeration and sedimentation (Sandri et al., 2014).

## Physiological Factors Influencing Rectal Drug Delivery

Rectal drug delivery can provide significant local and systemic levels for various drugs, despite the relatively small surface area of the rectal mucosa. However, the rectal route of administration can be affected by a number of physiological factors that will be discussed in this section. These factors should be considered in rectal formulation design, as they can affect drug bioavailability, efficacy, and safety.

### Anatomical Considerations

When developing rectal dosage forms for different age groups, it is important to consider the anatomical size difference between the rectum of adults and children (Jannin et al., 2014; Linakis et al., 2016). Any new formulations should be evaluated for bioavailability, efficacy, and safety in the target population. However, there are a few patient groups in which rectal dosage forms should be avoided or used with caution. In general, drugs are not commonly administered rectally in neonates (term or preterm), as it is associated with erratic absorption as well as a risk of damage to the delicate rectal lining that could lead to infection (Jannin et al., 2014). Similarly, the risk of trauma and subsequent infection with rectal dosage forms is also high for immunocompromized patients (Berlin et al., 1997).

In addition, it is anatomically easier for rectally administered drugs to reach the distal colon than the proximal colon. Drugs given by this route are typically formulated in solid dosage forms (e.g., suppositories) or in liquid/semi-liquid dosage forms (e.g., enemas and foams). In general, foams and suppositories are retained mainly in the rectum and sigmoid colon, while enema solutions have a greater spreading capacity (van Hoogdalem et al., 1991; Brown et al., 1997; Loew and Siegel, 2012). Enemas are able to spread over an area situated between the rectum and the splenic flexure, which is the sharp bend between the transverse colon and the descending colon (van Hoogdalem et al., 1991; Brown et al., 1997). Therefore, rectal administration of drugs for local action may be more suitable for conditions that affect the distal part of the large intestine, such as proctitis (inflammation of the lining of the rectum), hemorrhoids, and distal colitis.

### Site of Drug Absorption

The site of drug delivery in the rectum can affect the amount of the drug that reaches the systemic circulation. In general, drug absorption in the upper part of the rectum is transported to the liver *via* the portal system and thus undergoes first-pass metabolism, whereas drug absorption in the lower rectum is transported directly to the systemic circulation (de Boer et al., 1982; De Boer et al., 1984; Dujovny et al., 2004; Nunes et al., 2014; Purohit et al., 2018). This is of particular significance for drugs that have high hepatic clearance. However, it can be difficult to differentiate between the upper and lower regions when drugs are administered rectally. Anatomical differences in the venous drainage of the rectum between individuals can also significantly affect the amount of drug absorbed in the systemic circulation (de Boer et al., 1982; De Boer et al., 1984; van Hoogdalem et al., 1991). In addition, although systemic absorption cannot be completely avoided *via* rectal administration, limiting the amount of drug that is systemically absorbed is ideal for the treatment of local pathologies. Although a broad approximation, it has been reported that ∼50% of a drug that is absorbed from the rectum will bypass the liver, thus reducing the hepatic first-pass effect (De Boer et al., 1984; Brunton et al., 2018). However, wide variations of bioavailability can occur due to the aforementioned issues.

### Retention of the Formulation

For local or systemic drug absorption to occur, the formulation needs to be retained in the rectum for an adequate period of time. However, rectal formulations, particularly conventional dosage forms, can have problems with leakage, retention, and bloating (Allen et al., 2011). The contact time of the drug with the rectal mucosa is also important for absorption, as this will influence its bioavailability and efficacy. For absorption to occur, drugs need to be able to penetrate the mucus layer in order to reach the epithelial cells lining the rectum. Rectal mucus is mainly composed of water and mucins to form a fluid layer of ∼150 μm in thickness (range 75–250 μm) (Pullan et al., 1994; Johansson et al., 2013), with an estimated turnover time of 3–4 h (MacDermott et al., 1974; Nunes et al., 2014). This layer can act as a barrier for drug absorption.

### Fluid Volume and pH

The small fluid volume in the rectum and distal colon can affect rectal drug delivery. Compared to the small intestine, the volume of liquid in this region is significantly less, which may produce problems with the dissolution of some drugs (Jannin et al., 2014; Nunes et al., 2014; Purohit et al., 2018). As mentioned earlier, the pH in the rectum is typically considered neutral, which favors the absorption of drugs with pKa values near or above the physiologic range (Allen et al., 2011; Jannin et al., 2014; Nunes et al., 2014; Purohit et al., 2018). Changes in rectal pH can affect drug uptake by altering the ionization state of drugs. The rectal fluid has low buffering capacity, which means that administration of external products can significantly alter the pH in the rectum (Evans et al., 1988; Jannin et al., 2014; Nunes et al., 2014; Purohit et al., 2018). Variations in pH can impact on the absorption of drugs as well as lead to irritation or damage to the rectal mucosa (Allen et al., 2011; Nunes et al., 2014). These factors should be taken into account during formulation design to ensure efficient rectal drug delivery.

### Viscosity of Rectal Contents and Bowel Movements

The presence of stool in the rectum affects the viscosity of the rectal contents, which can subsequently affect drug dissolution, drug stability as well as contact of the drug with the mucosal wall for absorption (de Boer et al., 1982; van Hoogdalem et al., 1991; Nunes et al., 2014). These factors can lead to irregular drug absorption and non-specific interaction of drugs with fecal matter and mucus. Early expulsion of the drug, including following defecation, will also affect the concentration available to undergo passive absorption. Hence, it is important to consider the time of dosing with respect to an individual’s bowel movements (Sathyan et al., 2000). Frequency of bowel movements can be highly variable. For example, colonic transit times can vary significantly within and between individuals, with ranges from 6 to 70 h reported (Coupe et al., 1991; Rao et al., 2004). In addition, increased colonic motility in diarrhea can lead to reduced retention of rectal dosage forms and incomplete drug release (de Boer et al., 1982; van Hoogdalem et al., 1991; Nunes et al., 2014).

### Pathophysiological Factors Influencing Rectal Drug Delivery

Pathological conditions can influence the effectiveness of rectally administered drugs. This includes colorectal diseases such as inflammatory bowel disease (IBD), irritable bowel syndrome (IBS), hemorrhoids, anal fissures, bowel incontinence, and acute gastrointestinal infections. Variations in the amount of drug absorbed can occur with changes in tissue integrity, mucosal inflammation, and bowel motility. Conditions that affect the integrity and the barrier qualities of the rectal mucosa (e.g., local trauma, anal fissures, and ruptured hemorrhoids) can lead to increased drug absorption that can be difficult to predict as well as being painful to administer (Reinus and Simon, 2014). Likewise, mucosal inflammation can enhance epithelial permeability and, therefore, increase the amount of drug absorbed across the colorectal mucosa. For example, mucosal inflammation in IBD causes pathophysiological changes, including a disrupted intestinal barrier due to the presence of mucosal surface alterations, ulcers, and crypt distortions, as well as infiltration of immune cells (e.g., macrophages, lymphocytes, neutrophils, and dendritic cells) that promote inflammation (Li and Thompson, 2003; Antoni et al., 2014). Inflammation of the lining of the rectum (proctitis) can also occur in infections (e.g., sexually transmitted infections and gastrointestinal infections) and anal trauma (Hoque et al., 2012; Hatton et al., 2018).

Diseases that alter the motility of the gastrointestinal tract can also impact the effectiveness of rectally administered drugs by influencing retention, mucosal interaction, and the time available for disintegration, dissolution, and/or drug absorption. For example, diarrhea can occur in many acute gastrointestinal infections (Grover et al., 2008; Albenberg and Wu, 2014), in bowel incontinence (e.g., from muscle or nerve damage), and in chronic conditions such as IBD (Hua et al., 2015). Conversely, constipation is common in IBS and systemic pathologies that affect the endocrine system (e.g., hypothyroidism and diabetes) or central nervous system (e.g., multiple sclerosis and Parkinson’s disease) (Konturek et al., 2011; Hatton et al., 2019). Similarly, drugs that alter gastrointestinal motility can also affect rectal drug delivery (Watts et al., 1992; Brunton et al., 2018). This includes drugs that can cause constipation (e.g., opioids, anticholinergic agents, antidiarrheal agents, antacids containing aluminium or calcium, iron/calcium supplements, diuretics, verapamil, and clonidine) and drugs that can cause diarrhea (e.g., laxatives, antibiotics, colchicine, cytotoxic agents, digoxin, magnesium, NSAIDs, orlistat, acarbose, and metformin). Therefore, understanding the effect of disease comorbidities and co-administered drugs on gastrointestinal physiology is important when considering rectal drug formulations.

## Conventional Rectal Drug Delivery Approaches

Conventional rectal dosage forms can be categorized into three groups—liquid dosage forms (e.g., enemas), solid dosage forms (e.g., suppositories, capsules, and tablets), and semi-solid dosage forms (e.g., gels, foams, and creams). Rectal formulations have been developed to deliver drugs either locally or systematically and have been investigated to release the drug immediately or over a prolonged period of time (Purohit et al., 2018). The physicochemical properties of the drug (e.g., molecular weight, solubility, pKa, stability) and the required speed of absorption are important factors to determining which formulation to use (Jannin et al., 2014). For solid dosage forms, disintegration, liquefaction, and dissolution are required before drug absorption into the mucosa can occur. Therefore, absorption is generally slower from solid dosage forms compared to liquid dosage forms (Jannin et al., 2014; Purohit et al., 2018). This section will discuss the main conventional rectal dosage forms and the developments to improve their effectiveness for rectal drug delivery.

### Liquid Dosage Forms

Enemas are the main liquid dosage form for rectal drug delivery. They contain drugs in solution, suspension, or emulsion that are typically administered from disposable plastic squeeze bottles with an extended tip for rectal insertion. The solubility characteristics of the drug and additional solutes should be considered during pharmaceutical formulation, especially for solutions (Allen et al., 2011). Suspensions generally contain finely divided drug particles distributed throughout a vehicle in which the drug has minimal solubility. This formulation is particularly useful for drugs that are chemically unstable in solution (Allen et al., 2011). Emulsions are liquid preparations that have a dispersed phase composed of small globules of a liquid distributed throughout a vehicle in which it is immiscible. Emulsification enables the preparation of relatively stable and homogenous mixtures of two immiscible liquids (Allen et al., 2011). Enemas are mainly used to deliver drugs for the acute treatment of seizures, IBD, constipation, and as a bowel preparation for gastrointestinal diagnostic or surgical procedures (**Tables 1**, **2**).

There have been limited advances in the formulation of conventional enemas. Self-emulsifying drug delivery systems (SEDDS) were developed as a means to improve the bioavailability of poorly soluble drugs (Cherniakov et al., 2015). In general, SEDDS are composed of an oily base and a surfactant, with or without a hydrophilic co-solvent or cosurfactant. This creates a liquid dosage form that transitions to an oil-in-water emulsion once in contact with the aqueous phase at the site of administration. SEDDS are thought to improve bioavailability by enhancing drug solubility and improving membrane permeability (Cherniakov et al., 2015). Although more commonly studied for the oral route of administration (Gupta et al., 2013; Cherniakov et al., 2015; Karamanidou et al., 2016), the advantages of SEDDS have shown promise for rectal drug delivery (Kim and Ku, 2000; Kauss et al., 2018). For example, Kauss et al. (2018) evaluated the use of SEDDS to improve the systemic bioavailability of ceftriaxone for potential use as a rectal antibiotic therapy in neonates. *In vivo* results in rabbits showed rapid absorption of ceftriaxone in the SEDDS formulation following rectal administration, achieving 128% bioavailability compared to rectally delivered ceftriaxone capsule (powder control formulation).

The properties of the enema itself can influence rectal drug delivery. Maisel et al. (2015) demonstrated that the composition of the enema can determine whether drugs are delivered locally or systemically. In particular, strongly hypotonic (absorption-inducing) and hypertonic (secretion-inducing) enemas caused rapid systemic drug uptake, whereas moderately hypotonic enemas (with ion compositions similar to feces) resulted in high local tissue levels with minimal systemic drug absorption. Interestingly, hypertonic enemas caused extensive epithelial tissue damage in the colorectal region, which promoted systemic drug absorption. Hypotonic enemas, however, caused no detectable epithelial damage. Strongly hypotonic enemas were suggested to transport drug through the epithelium by both transcellular and paracellular fluid absorption to the systemic circulation. Moderately hypotonic enemas were able to flow through the mucus layer and increase local drug bioavailability in the colorectal tissues, but they were mild enough to minimize systemic absorption.

Further study is required to determine the safety of various enema ion compositions in humans and for repetitive use (Maisel et al., 2015). In addition, the interaction of specific drugs in liquid dosage forms with the colorectal mucosa can also impact on the efficiency of drug absorption, and therefore, should be comprehensively evaluated. The use of liquid dosage forms generally allow faster absorption since drug release and dissolution issues are usually circumvented (Allen et al., 2011). However, the volume administered can affect drug retention in the rectum. For example, smaller volumes have been shown to have greater retention, while volumes higher than 80 ml can stimulate defecation (van Hoogdalem et al., 1991; Nunes et al., 2014). All conventional liquid dosage forms can suffer from various degrees of leakage, which can lead to irregular drug absorption.

### Solid Dosage Forms

Suppositories are the most common rectally administered dosage form used clinically. They are solid dosage forms containing drugs that are either dispersed or dissolved in a suitable base (Allen et al., 2011). Drugs are typically mixed with the suppository excipients during manufacturing to form a homogenous system. Suppositories are generally composed of either a lipophilic base (e.g., cocoa butter, coconut oil, hydrogenated vegetable oils, and hard fats) or hydrophilic base (e.g., glycerinated gelatin and polyethylene glycols) (Allen et al., 2011; Jannin et al., 2014; Ham and Buckheit, 2017). Lipophilic bases are immiscible with body fluids and readily melt at body temperature to release the drug on the mucosal surface, whereas hydrophilic bases need to dissolve in the physiological fluids for drug release (Allen et al., 2011; Jannin et al., 2014; Ham and Buckheit, 2017).

Suppositories can be designed to have different rates and degrees of drug release for absorption. In particular, the composition of the suppository base, including the use of surfactants or other additives, and the physicochemical properties of the drug (e.g., solubility and particle size) can confer different drug release profiles (Nunes et al., 2014; Leppik and Patel, 2015). For drugs that are dissolved (soluble) in the suppository base, drug release occurs as the suppository dissolves or melts onto the mucosal surface where the drug molecules then diffuse out. For drugs that are dispersed (insoluble) in the suppository base, the opposing solubility properties encourage the drug to leave the dosage form and then begin solubilizing in the physiological fluid (Jannin et al., 2014; Ham and Buckheit, 2017). Therefore, hydrophilic drugs tend to show better release in lipophilic bases, and lipophilic drugs have better release in hydrophilic bases. In this case, particle size of the drug will also influence the rate of absorption and bioavailability (Leppik and Patel, 2015).

Despite the advantages of conventional suppositories for rectal drug delivery, they are associated with issues such as irregular drug absorption, leakage, and discomfort. Several advances have been made to improve on bioavailability, formulation retention, and patient acceptability of these solid dosage forms. This includes the addition of surfactants (e.g., polysorbate 80, Tween 20, and Span 60) to either the hydrophilic or lipophilic phase of the formulation to create solid emulsion-type suppositories (Abd el-Gawad et al., 1988; Gugulothu et al., 2010; Abou el Ela Ael et al., 2016). Emulsion bases were reported to have higher rates of drug release compared to lipophilic and hydrophilic suppository bases (Abd el-Gawad et al., 1988; Jannin et al., 2014; Abou el Ela Ael et al., 2016). The presence of surfactants in the formulation improved the wettability of the suppository base matrix, thereby enhancing the release and dissolution of the embedded drug particles (Abd el-Gawad et al., 1988; Jannin et al., 2014; Abou el Ela Ael et al., 2016).

Hollow-type suppositories have been developed and modified to enhance the absorption of various drugs (Watanabe et al., 1986; Watanabe et al., 1986; Matsumoto et al., 1989; Uekama et al., 1995; Watanabe et al., 1998; Kowari et al., 2002; Kaewnopparat et al., 2004; Shiohira et al., 2009). This type of suppository essentially contains a hollow space in the center that is filled with the drug in solid, liquid, or semi-solid form. The solid outer shell of the suppository can be composed of hydrophilic or lipophilic base materials and can incorporate other constituents to confer additional release properties, such as mucoadhesion and sustained release. This design has the benefits of controlling the dose of the drug, allowing convenient interchangeability of the drug, and preventing any interactions between the drug and the base material.

Furthermore, dimple-type suppositories were developed by Matsumoto et al. (2017) to improve the rectal delivery of poorly absorbable drugs such as peptides and oligonucleotides. These suppositories have one or more dimples on the surface where drugs are embedded. It was proposed that concentrating the drug to a limited area on the surface of the suppository would lead to a higher rate of drug release and absorption when administered into the rectum. In addition, limiting the drug concentration toward the surface of the suppository increases its contact with the rectal mucosal surface and creates a concentration gradient for passive absorption of the drug across the mucosa. Interestingly, *in vitro* release studies showed that the time to 50% drug release was dependent on the melting point of the lipid used for sealing the dimples and not on the number of dimples (Matsumoto et al., 2017).

Additional studies are required to comprehensively evaluate the pharmacokinetics, efficacy, and safety of drugs formulated in the different suppository dosage forms in humans for both local and systemic absorption. Evaluations should also be compared between single dose and multiple dose therapies to ensure reproducibility of the results. This data will determine the clinical translatability of the formulations.

### Semi-Solid Dosage Forms

Gels and foams are the most common semi-solid dosage forms used for rectal drug delivery. These formulations generally require the use of an applicator that has to be filled with the drug formulation prior to dose administration (Allen et al., 2011). Rectal gels are a semi-solid formulations that contain a solvent trapped within a polymer network to create a viscous consistency. Viscosity of the gel can be modified by the addition of co-solvents (e.g., glycerin and propylene glycol) and electrolytes (Allen et al., 2011; Nunes et al., 2014). They are easy and inexpensive to manufacture, however can suffer from stability issues, leakage, and messiness upon administration. The spreading features of rectal gel formulations are highly dependent on properties such as mucoadhesion and viscosity (Allen et al., 2011; Nunes et al., 2014). These properties can also affect the site of drug delivery and the fraction that undergoes hepatic first-pass metabolism.

One of the main advancements in conventional rectal dosage forms is the development of liquid suppositories, which more closely resemble semi-solid dosage forms rather than solid dosage forms. This includes the development of liquid suppositories containing thermosensitive polymers (Miyazaki et al., 1998; Fakhar Ud and Khan, 2019), mucoadhesive polymers (Koffi et al., 2008; Ye et al., 2016; Xu et al., 2017; Shi et al., 2019), or a combination of thermosensitive and mucoadhesive polymers (Choi et al., 1998; Yun et al., 1999; Ryu et al., 1999; Koffi et al., 2008; Barakat, 2009; Lo et al., 2013; Liu et al., 2018; Akl et al., 2019). Poloxamers are the most commonly used thermosensitive polymers in pharmaceutical formulation. They are nontoxic amphiphilic molecules that exhibit reverse thermal gelation. This allows them to remain in a liquid state at room temperature and convert into a gel consistency at body temperature, thereby allowing ease of administration into the body, reduced leakage, restricted spreading in the rectal cavity, and improved contact with the rectal mucosal surface (Yong et al., 2001; Barakat, 2009; Akl et al., 2019). Poloxamer molecules form small micellar units at room temperature and large micellar cross-linked network at body temperature (Akl et al., 2019). However, poloxamer gels on their own can have inadequate mucoadhesion, weak mechanical strength, and high permeability to water (Yong et al., 2001; Barakat, 2009; Akl et al., 2019).

Mucoadhesive polymers (e.g., carbopol, sodium alginate, polycarbophil, hydroxypropyl methylcellulose, hydroxyethyl cellulose, and methylcellulose) have been used in combination with thermosensitive polymers to improve gel strength and mucoadhesion. For example, the carboxyl groups in the mucoadhesive polymers can bind strongly with the cross-linked poloxamer gel, thereby positioning its molecules in between the gel to enhance overall strength (Barakat, 2009; Akl et al., 2019; Fakhar Ud and Khan, 2019). In addition, mucoadhesion is enhanced by hydrogen bonding of the polymers with the oligosaccharide chains of the rectal mucosal layer through hydroxyl and carboxyl groups (Lehr et al., 1990; Qi et al., 2006; Barakat, 2009; Akl et al., 2019). The enhanced mucosal retention of these hydrogels promotes improved drug release and absorption. It should be noted that cellulose ether polymers (e.g., hydroxypropyl methylcellulose, hydroxyethyl cellulose, and methylcellulose) also possess controlled release characteristics. These hydrogels are able to swell over time, which would also allow the encapsulated drug to be released at a continuous rate (Vueba et al., 2006; Barakat, 2009; Shi et al., 2019).

Foams are generally considered a colloidal dosage form, with a hydrophilic liquid continuous phase containing a foaming agent and a gaseous dispersion phase distributed throughout (Allen et al., 2011). Following rectal administration, they transition from a foam state to a liquid or semi-solid state on the mucosal surface. The structure of the foam is affected by parameters such as concentration and nature of the foaming agent, pH and temperature of the system, and viscosity of the liquid phase (Arzhavitina and Steckel, 2010). Foaming agents are amphiphilic substances that are important for foam generation and stabilization. The molecules contain hydrophilic components that are soluble in the aqueous phase and hydrophobic components that form micelles to minimize contact with the aqueous phase (Arzhavitina and Steckel, 2010). Rectal foams are mostly aerosol foams that are formulated to treat anorectal inflammation (e.g., hemorrhoids and anal fissures) and distal proctocolitis (e.g., distal ulcerative colitis) (Campieri et al., 1992; Lee et al., 1996; Arzhavitina and Steckel, 2010; Loew and Siegel, 2012; Sandborn et al., 2015). The advantages of foams for rectal drug delivery include convenient administration with minimal discomfort and leakage. Despite these advantages, there are not many rectal foam formulations that are commercially available. This is partly due to the issues with foam stabilization, accuracy of the dose administered, and irregular drug absorption (Arzhavitina and Steckel, 2010). Developments in this area have included the addition of mucoadhesive polymers to improve the retention of the formulation with the rectal mucosa for drug absorption (Arzhavitina and Steckel, 2010; Petkova et al., 2012; Politova et al., 2012).

## Nanoparticulate Rectal Drug Delivery Approaches

Incorporation of nanoparticulate systems into rectal dosage forms has been investigated to improve the therapeutic effectiveness of drugs for both local and systemic therapy. Nanoparticulate rectal dosage forms differ from conventional rectal dosage forms by encapsulating or loading the drug into nanoparticles prior to dispersion in a formulation base (e.g., gel, suppository, and enema). From a pharmaceutical perspective, nanoencapsulation allows the ability to improve the solubility of hydrophobic compounds, modify drug release kinetics (e.g., controlled release or sustained release), and protect compounds that are sensitive to degradation (Shajari et al., 2017; Hua et al., 2018; Mesquita et al., 2019). From a biological perspective, nanoparticulate systems confer the following advantages: (i) improve cellular uptake into mucosal tissues and cells, (ii) promote accumulation to the site of mucosal disease (e.g., inflamed tissues), (iii) prolong residence time of encapsulated compounds (even when colonic motility is increased in diarrhea), and (iv) enable easier transport in the gastrointestinal tract to provide more uniform distribution and drug release within the colorectal region (Hua et al., 2015; Zhang et al., 2017; Hua et al., 2018; Mesquita et al., 2019).

For nanoparticulate dosage forms to be effective for rectal drug delivery, two main factors should be considered. First are the physicochemical properties of the nanoparticles (e.g., size, charge, composition, and surface properties) for optimal interaction with the rectal or colorectal mucosa. These properties can promote better contact with the mucosal surface for improved mucosal penetration, cellular uptake, and drug release (Hua et al., 2015; Zhang et al., 2017). Second is the interaction of the nanoparticles with the formulation base. The nanoparticles should be stable when incorporated into the pharmaceutical base, especially during manufacturing and storage. In addition, the formulation base should increase the retention of the formulation in the rectum, without impeding the transport and interaction of the nanoparticles with the mucosal tissue. Adhesion to the mucosa is a requirement for effective rectal drug delivery, as it reduces the clearance of nanoformulations by mucus, leakage, or rapid transit time (e.g., diarrhea) (Mesquita et al., 2019). A number of different nanoparticulate systems have been evaluated for rectal drug delivery. This section will discuss the effectiveness of each of the systems in terms of the formulation base.

### Nanoparticulate Liquid Dosage Forms

Liquid dosage forms are typically used in initial studies to evaluate the potential of nanoparticulate systems for rectal drug delivery. This is likely due to convenience, as the nanoparticles are usually manufactured in aqueous liquid such as water and buffered solutions (Mesquita et al., 2019). In addition, it is common for proof-of-concept studies to be evaluated in aqueous liquid to minimize the interference of the formulation base with the nanoparticles. This is evident in a large portion of studies focused on colon-targeted drug delivery, whereby nanoparticles are administered rectally to determine efficacy and safety early on in animal models (Lamprecht, 2010; Hua et al., 2015; Maisel et al., 2015; Zhang et al., 2017) and humans (Schmidt et al., 2013), prior to the added complexities of formulation design for clinical translation. For example, Maisel et al. (2015) evaluated the effect of surface chemistry on nanoparticle interaction and distribution in the gastrointestinal tract following oral and rectal administration in healthy mice and in a mouse model of ulcerative colitis. Various nanoparticle sizes (40, 100, 200, and 500 nm) were also assessed. The study showed that nanoparticles coated with polyethylene glycol (PEG) of all sizes were able to be efficiently distributed over more of the colorectal tissue surface in both healthy mice and mice with TNBS-induced colitis, which is likely to provide improved drug delivery for both local and systemic applications. Surface PEGylation of nanoparticles creates a hydrophilic surface chemistry that reduces interaction of the nanoparticles with the gastrointestinal environment and confers mucus-penetrating properties (Cu and Saltzman, 2008; Lai et al., 2009; Tang et al., 2009; Hua et al., 2015).

There are fewer studies focused on nanoparticulate drug delivery in a liquid dosage form to specifically the rectal mucosa for local and/or systemic action (das Neves et al., 2013; Kamel et al., 2013; Schmidt et al., 2013; Maisel et al., 2015; Nunes et al., 2018). Of these studies, Schmidt et al. (2013) were the first to investigate the potential of conventional nanoparticle (mean particle size of 250 nm) and microparticle (mean particle size of 3 µm) uptake into the rectal mucosa of humans with and without IBD. Both poly(lactic-co-glycolic acid) (PLGA) nanoparticles and microparticles were dispersed in saline solution containing 10% human albumin. The addition of the protein in the dispersion medium sterically stabilized the particles and reduced their surface charge by adsorption to the particle surface. The results showed accumulation of microparticles in ulcerous lesions of patients with both rectal Crohn’s disease and ulcerative colitis. There was a clear size-dependent difference regarding the accumulation of particles in IBD patients, with nanoparticles only detectable in traces in the mucosa of these patients. The study demonstrated that microparticles exhibited accumulation and bioadhesion to the inflamed mucosal wall; however, no absorption across the epithelial barrier was detected. Conversely, nanoparticles were translocated to the serosal compartment of IBD patients, possibly leading to systemic absorption. In healthy control patients with the rectal mucosal surface intact, nearly no nanoparticles or microparticles were visible. The study suggested that nanoparticles might not be required for local drug delivery to intestinal lesions in humans. However, the reason for the discrepancy of particle size between animal and human studies is unclear. It should be noted that, while particle accumulation in ulcerated areas was statistically significant, the total fraction of particles penetrating into the rectal mucosa was relatively low in the study.

Liquid bases at physiological pH and osmolality are commonly used for rectal drug delivery. However, Maisel et al. (2015) showed that the composition of enemas can be optimized for the local and/or systemic delivery of nanoparticles. Hypotonic sodium-based enemas were shown to be an ideal liquid formulation base to enhance the distribution of PEGylated polystyrene nanoparticles (mean particle size of 60 and 230 nm) on the colorectal epithelial surface in comparison to isotonic and hypertonic enemas and potassium-based enemas. In particular, hypotonic sodium-based enemas induced fluid absorption that promoted uniform nanoparticle distribution over the epithelial surface, whereas hypertonic sodium-based enemas caused fluid secretion and bowel distension that prevented nanoparticles from being in close contact with the mucosal surface. Although when used as a pretreatment, strongly hypertonic enemas were able to damage the colorectal epithelium, which allowed penetration of the hypotonically delivered nanoparticles into the tissue.

Overall, nanoparticulate liquid dosage forms would still have the same issues as conventional liquid dosage forms that were mentioned earlier. Although liquid dosage forms have greater spreading capacity in the rectum, they can suffer from low retention and leakage—both of which can lead to irregular drug absorption. Their use will be highly dependent on the clinical application and frequency of dosage. Importantly, the initial results from liquid dosage forms support the potential of nanoparticles (and microparticles) for improving rectal drug delivery.

### Nanoparticulate Solid Dosage Forms

There are only a few studies which have incorporated nanoparticles into solid dosage forms for rectal drug delivery. Abdelbary et al. (Abdelbary and Fahmy, 2009) developed solid lipid nanoparticles (SLN) containing the water-insoluble drug, diazepam, to confer both rapid onset of action and prolonged drug release for the potential acute management of severe seizures. Results showed that varying the concentration or type of lipid matrix or surfactant affected the particle size, entrapment efficiencies, and release profiles of the nanoparticles. Transmission electron microscopy and laser diffractometry studies revealed that 60% of the formulations had particle sizes less than 500 nm. The nanoparticles were effectively incorporated into suppositories composed of hard fats (Witepsol W35 and Witepsol S58). *In vitro* studies showed significantly prolonged drug release from the SLN-containing suppositories in comparison to suppositories containing free drug (control). However, drug release from the control formulation was significantly faster than the SLN-containing suppository formulations. Further investigation is necessary to determine the efficacy of the rectal formulations *in vivo*. The release profile of the diazepam-loaded SLNs in a primarily hydrophilic base would also be of interest, as this may allow a faster release of drug into the physiological fluid that would be beneficial in emergency clinical applications.

This concept of having opposing solubility properties of the drug-loaded nanoparticles and suppository base was investigated by Mohamed et al. (2013). This study incorporated the hydrophilic drug, metoclopramide, into SLNs (particle size range of 24.99–396.8 nm) that were then formulated into suppositories with a lipophilic base. Suppositories containing a cocoa butter base demonstrated the highest release of metoclopramide from SLNs, which was likely due to it having a lower melting point and its lack of hydrophilicity. The formulation also demonstrated sustained release of the drug due to coating with lipids in the nanoparticles. In particular, metoclopramide-loaded SLN suppositories produced the same gastric emptying percentage as the marketed metoclopramide suppository (Primperan) with additional sustained release characteristics *in vivo*, thereby avoiding the need for multiple dosing.

Similarly, Siczek et al. (2018) used cocoa butter suppositories to validate the possibility of effectively releasing silver from silver-coated glass beads for local anti-inflammatory action in conditions such as IBD. It should be noted that the borosilicate glass beads had an initial diameter of 1,000 µm before coating with silver. *In vitro* drug release assays of the silver-coated glass beads showed rapid release of silver, with nearly half of the amount of the deposited metal being released in the first 30 min of incubation. After 24 h, approximately 30% of the silver remained on the glass beads. Further studies are still needed to evaluate the rate of silver release from silver-coated glass beads from the suppository as well as the effectiveness of the formulation *in vivo*. Analysis of the prepared suppositories containing silver-coated glass beads using X-ray CT demonstrated an effective method to attain homogenous distribution of the beads in the entire volume of the suppository with minimal sinking or agglomeration.

Despite there being only a few studies to date that have incorporated nanoparticles into solid dosage forms for rectal drug delivery, the basis for further investigation is warranted. In particular, the *in vitro* data and initial *in vivo* data show potential of the formulation strategy in terms of drug release profiles. Proof-of-concept studies are still required to demonstrate the bioavailability, efficacy, and safety of these nanoparticulate formulations.

### Nanoparticulate Semi-Solid Dosage Forms

Gels are the most likely of the formulation bases to have translational potential for the delivery of nanoparticles rectally. The viscous consistency of gels promotes improved retention of formulations in the rectum and enhances contact with the rectal mucosa for drug absorption. As mentioned previously for conventional semi-solid dosage forms, a number of advances have been made for rectal drug delivery with the use of mucoadhesive polymers and/or thermosensitive polymers in the formulation base. The choice of gel base and its composition are dependent on the physicochemical properties of the nanoparticles and ideally should not interfere with drug release from the nanoparticles or the interaction of nanoparticles with the rectal mucosa.

Mucoadhesive bases alone have not been evaluated for nanoparticle delivery into the rectum. Instead, the use of thermosensitive polymers in the formulation base has been more common for the rectal delivery of nanoparticles (Seo et al., 2013; Din et al., 2015; Din et al., 2017; Melo et al., 2019). These polymers create an initial liquid dosage form at room temperature that allows ease of administration and mucosal spreading, before transitioning to a gel phase at body temperature. Melo et al. (Melo et al., 2019) investigated the colorectal distribution and retention of PLGA nanoparticles (mean particle size of 170–180 nm) incorporated into a thermosensitive base (poloxamer 407). *In vitro* drug release assays of dapivirine loaded into this nanoparticle formulation showed faster and overall higher drug release over 8 h in comparison to free drug in thermosensitive base and dapivirine-loaded nanoparticles in PBS. In addition, *in vivo* studies in mice indicated that the thermosensitive base exhibited slower but wider distribution of the nanoparticles in the colorectal region. Enhanced retention of the nanoparticles was also evident in the colorectum.

Similarly, Din et al. (2017) developed a novel rectal formulation of irinotecan for the local treatment of rectal cancer. This nanoparticulate dosage form consisted of thermosensitive irinotecan-encapsulated SLNs (mean particle size of 190 nm) dispersed in a thermosensitive poloxamer solution to create a double-reverse thermosensitive nanocarrier system (DRTN). Therefore, the formulation base transitions from a liquid to a gel state after rectal administration, whereas the SLNs are composed of lipids that are solid at 25°C and melt at body temperature. The DRTN dosage form showed sustained drug release with minimal burst effect and a relatively constant plasma concentration of irinotecan at 1–3 h in healthy rats. Interestingly, *in vivo* evaluation in tumor xenograft athymic nude mice showed significant decreases in tumor volume with both DRTN and the control hydrogel (irinotecan in thermosensitive base) in comparison to intravenous irinotecan solution. Histopathological analysis suggested that DRTN had significantly improved anti-tumor activity compared to both controls due to its sustained plasma concentrations.

The combination of thermosensitive and mucoadhesive polymers in the formulation base for the rectal delivery of nanoparticles has not been extensively examined. Moawad et al. (2017) developed nanotransfersomes (mean particle size of 150 nm) that were incorporated into a formulation base containing poloxamer 407 (thermosensitive polymer) and hydroxypropyl methylcellulose (mucoadhesive polymer) to improve the bioavailability of tizanidine (myotonolytic drug). *In vivo* pharmacokinetic studies in rabbits showed enhanced drug bioavailability by approximately 2.2-fold and 1.4-fold for nanotransfersome gel and free drug in gel, respectively, in comparison to oral drug solution. This enhancement in bioavailability was likely to be due to the partial avoidance of hepatic first-pass metabolism by the rectal route. Higher bioavailability of the nanotransfersome gel was also attributed to the permeation enhancing effect of the nanoparticles. In addition, both rectal formulations significantly increased the half-life of tizanidine (10.13 h for nanotransferome gel and 7.21 h for free drug gel) compared to oral drug solution (3.41 h). The results suggest that the use of the thermosensitive-mucoadhesive gel base as well as nanoparticulate encapsulation of the drug both delayed the release of tizanidine to produce a sustained release effect.

The limited studies to date support the use of semi-solid dosage forms for the rectal delivery of nanoparticles. Further studies are needed to determine the interaction of the semi-solid dosage forms on the nanoparticulate systems, including: (i) distribution following rectal administration, (ii) retention of the formulation in the rectum, (iii) adhesion and/or uptake of nanoparticles in the rectal mucosa, (iv) movement of nanoparticles in the semi-solid dosage form, (v) drug release from nanoparticles entrapped in the semi-solid dosage form, and (v) stability of the formulation during manufacturing and storage.

## Rectal Formulations Approved and in Clinical Trials

A number of rectal formulations are on the market with more in clinical development. **Tables 1** and **2** show examples of the rectal formulations that are clinically approved for local absorption and systemic absorption, respectively. These formulations generally contain drugs that have a wide therapeutic window between the concentration that causes therapeutic effects and the concentration that causes toxicity. This allows a safe margin that accounts for the variability in rectal drug absorption. Approved rectal formulations are typically indicated for conditions that require short-term therapy. Exceptions include a few locally acting formulations, such as mesalazine or corticosteroids that are used for a longer duration to induce remission in patients with ulcerative proctitis or ulcerative proctosigmoiditis that occurs in IBD. Clinical studies have demonstrated budesonide rectal foam and enema to be efficacious in these conditions, while reducing the risk of systemic steroid-related adverse effects (Gross et al., 2006; Sandborn et al., 2015).

The majority of the rectal formulations in clinical trials (**Table 3**) incorporate already approved drugs or novel compounds into conventional rectal dosage forms—in particular, suppositories, enemas, and rectal gels. Many of these formulations are still in the early clinical phases of investigation and are indicated for local pathologies, including hemorrhoids, constipation, bowel preparation, anal fissure, IBD, fecal microbiota transplant, and infections (e.g., HIV prevention). The very few that are focused on systemic drug absorption with rectal formulations are for the treatment of pain. Similar to the approved rectal formulations, those in clinical trials are predominantly used for short-term therapy. Furthermore, innovative rectal dosage forms, such as nanoparticulate systems, have yet to reach the clinical development phase. Thermosensitive rectal gels are likely the most innovative platform in clinical trials. They have been evaluated for parameters such as safety, preference, distribution, and retention in healthy patients as well as in patients with ulcerative colitis. With further advances in rectal drug formulation and comprehensive preclinical evaluation, we should expect to see more progressing to clinical studies.

**Table 3:** Rectal formulations in clinical trials *(Ref: clinicaltrials.gov)*.

Drug	Dosage form	Indication	Status
Ceftriaxone	Suppository	Healthy	Phase I
Quetiapine	Suppository	Dementia, delirium	Phase I completed
Ibuprofen	Suppository	Healthy	Phase I completed
NRL001	Suppository (slow release)	Incontinence	Phase I completed
Hydrocortisone	Suppository, enema	Healthy	Phase I completed
Nifedipine	Suppository	Chronic anal fissure	Phase I/II completed
Hydrocortisone	Suppository	Internal hemorrhoids	Phase II completed
Diclofenac	Suppository	Carcinoma prostate	Phase II completed
Meloxicam	Suppository	Ankylosing spondylitis	Phase III completed
Balsalazide	Suppository, enema	Ulcerative colitis	Phase III completed
Belladonna + opium	Suppository	Nephrolithiasis	Phase IV completed
Belladonna + opium	Suppository	Post-partum pain	Phase IV completed
Belladonna + opium	Suppository	Post-operative pain	Completed
Fluocortolone + lidocaine	Suppository, cream	Acute hemorrhoids	Not stated
Nil	Suppository, enema and rectal insert	HIV prevention	Not stated
Tenofovir	Enema	HIV prevention	Phase I
Fecal microbiota	Enema	Infection due to resistant organism	Phase I
Fecal microbiota	Enema	Crohn’s Disease	Phase I
Fecal microbiota	Enema	Acute pancreatitis	Phase I
Mesalamine	Enema	Healthy	Phase I completed
ALTH12	Enema	Ulcerative colitis	Phase I completed
TF037	Enema	Colonoscopy	Phase I completed
SB012	Enema	Ulcerative colitis	Phase I/II completed
Fecal microbiota	Enema	Severe acute malnutrition	Phase I/II
Fecal microbiota	Enema	Clostridium difficile infection	Phase II
Fecal microbiota	Enema	Ulcerative colitis	Phase II completed
PUR0110	Enema	Left-sided ulcerative colitis	Phase II completed
Manuka honey	Enema	Pouchitis	Phase II completed
Promelaxin	Enema	Chronic functional constipation	Phase IV
Chloral hydrate	Enema	Congenital cataract	Completed
NER1008	Enema	Colorectal cancer	Completed
IQP-0528	Rectal gel	HIV prevention	Phase I
Lidocaine	Rectal gel	Hemorrhoids	Phase I
Nil	Rectal gel (thermosensitive)	Ulcerative colitis	Phase I completed
Tenofovir	Rectal gel (mucoadhesive)	HIV prevention	Phase I completed
Tenofovir	Rectal gel (mucoadhesive)	HIV infection	Phase I completed
Dapivirine	Rectal gel	HIV infection	Phase I completed
PC-1005	Rectal gel	HIV infections	Phase I completed
Maraviroc	Rectal gel	HIV/AIDS	Phase I completed
PP110	Rectal gel	Bleeding hemorrhoids	Phase II/III completed
Lidocaine + diclofenac	Rectal gel	Anal fissure	Phase IV completed
Nil	Rectal gel (thermosensitive)	Healthy	Completed

## Considerations for Translational Development

The rectal route of administration has significant advantages for both the local and systemic delivery of drugs. However, there has been a general lack of research in this important area of drug formulation when compared to other routes for gastrointestinal drug delivery. In particular, there is a need for comprehensive studies on the biological interactions of rectal drug delivery in both adults and children, as well as continued innovations in rectal drug formulations.

From a biological perspective, there should be comprehensive analysis of the *in vivo* fate and interactions of drugs delivered in existing and new rectal dosage forms with the blood, tissue, cellular, and intracellular compartments in both healthy and diseased states (Nehoff et al., 2014; Sercombe et al., 2015; Hare et al., 2017; Hua et al., 2018). This includes pharmacokinetics, stability, permeability, efficacy, and safety of the formulation. Attention should also be given to the performance of these dosage forms in the heterogeneous nature of the human gastrointestinal environment (Hua et al., 2015; Zhang et al., 2017). As discussed earlier, rectal drug delivery can be affected by a number of physiological factors, which can lead to wide variations in the amount of drug absorbed. This is particularly problematic for drugs with a narrow therapeutic index or serious conditions that require predictable drug levels. Therefore, use of the rectal route of administration is unlikely to be clinically feasible in these situations.

In addition, there are an increasing number of studies investigating the potential of rectal drug delivery for the treatment of more chronic conditions, including diabetes (Matsumoto et al., 2017; Shi et al., 2019), infections (das Neves et al., 2013; Ham and Buckheit, 2017; Nunes et al., 2018), hypertension (Abou el Ela Ael et al., 2016), asthma (Shiohira et al., 2009), chronic anal fissure (Ivanova et al., 2019), and cancer (Lo et al., 2013; Seo et al., 2013; Ye et al., 2016; Din et al., 2017). Although encouraging results were reported in these studies, with many designed for sustained release activity, they generally did not evaluate the formulations over a long study period. Further studies are required to assess the reproducibility and variation in the pharmacokinetics, efficacy, and safety of these formulations for long-term dosing. Dosing frequency of rectal formulations will also be a major factor for clinical translation, with once daily dosing providing better patient compliance.

For innovative platforms, such as nanoparticles, safety of the different carriers following uptake needs to be explored further, including both acute and chronic toxicity (Nystrom and Fadeel, 2012; Accomasso et al., 2018). Studies focused on the toxicology of these delivery systems in the human gastrointestinal tract have been limited and is likely to vary according to the particle size and composition (Bergin and Witzmann, 2013; Talkar et al., 2018; Vita et al., 2019). The pace for the clinical translation of nanoparticulate dosage forms has been relatively slow as the development trajectory is very costly, complex, and time-consuming (Hua et al., 2018). There has to be a clear positive benefit-to-risk ratio that will accompany the use of nanoparticles for rectal drug delivery, especially when compared to an approved counterpart or existing therapies (Hua et al., 2018). Therefore, *in vivo* evaluation of innovative platforms should be compared with appropriate control formulations to provide meaningful data on the influence of the drug, carrier, and/or formulation base for effective rectal drug delivery (Hua et al., 2018).

Furthermore, the evaluation of many of the rectal dosage formulations has been limited to *in vitro* (e.g., drug release and cellular uptake) and/or *ex vivo* (e.g., mucoadhesion) studies. Therefore, caution should be taken when interpreting the data, as the same effect in animals or humans cannot be guaranteed. Use of rodents for *in vivo* studies can also have its limitations for examining rectal drug delivery for clinical use. For example, the anatomy and physiology of rodents can affect the distribution of the dosage form as well as the amount of the formulation that can be administered rectally (Melo et al., 2019). In comparison to humans, rodents tend to defecate more frequently, have more intense bowel movements, and have faster turnover of mucus in the rectum (Mule et al., 2010; Ermund et al., 2013; Padmanabhan et al., 2013; Melo et al., 2019). These factors should be taken into account when designing *in vivo* studies. Although assessment in larger animal models with similar gastrointestinal transit times to humans (e.g., pigs and dogs) would be more applicable for clinical translation (Kararli, 1995; Maisel et al., 2015), this is generally not feasible and is associated with its own ethical considerations. Therefore, previous studies have suggested an alternate time scale to evaluate colorectal retention of drug formulations administered rectally in rodents, with 15 min, 2 h, and 6 h corresponding to short, medium, and long retention times, respectively (Maisel et al., 2015; Nunes et al., 2018; Melo et al., 2019).

From a commercial development point of view, the complexity in the design and development of rectal dosage forms also needs to be minimized as much as possible, to create dosage forms that are able to be reproducibly prepared and characterized (Hua et al., 2018). The pharmaceutical characterization of different rectal dosage forms has been comprehensively addressed in other reviews (Jannin et al., 2014; Nunes et al., 2014; Purohit et al., 2018) and is an important consideration for translational development. This includes the availability of appropriate testing methods and standardized protocols for quality control that meet regulatory requirements. For example, rectal formulations should be physically and chemically stable after the manufacturing process, during long-term storage, and upon clinical administration to ensure reproducible release kinetics. In addition, rectal dosage forms should be tailored for use in adults and children, with the latter also having further anatomical size differences and dose requirements that should be taken into consideration (Jannin et al., 2014; Linakis et al., 2016). Ideally, they should deliver single doses to provide reproducible bioavailability, efficacy, and safety. Other considerations include potential for scale-up for large-scale manufacturing, availability of materials and industrial equipment, and overall cost of dosage form development (Hua et al., 2018; Purohit et al., 2018). As mentioned for nanoparticulate formulations, there also needs to be a clear benefit of efficacy and/or safety with any new drug formulation compared to clinically available dosage forms for clinical translation to be justified (Hua et al., 2018).

## Conclusion

The rectal route for drug delivery is still relatively underutilized despite its advantages. Although the oral route is the most convenient and preferred route for drug administration, there are a number of circumstances that have been discussed where this is not possible from either a clinical or pharmaceutical perspective. In these cases, the rectal route may represent a practical alternative and can be used to administer drugs for both local and systemic action. Continued innovations in rectal drug formulation and comprehensive studies on the biological interactions of rectal drug delivery are required to fully exploit the potential of this route to treat systemic and local diseases.

## Author Contributions

SH was involved in conception of the idea for the review, drafted the manuscript, and approved the final version of the manuscript.

## Conflict of Interest

The author declares that the research was conducted in the absence of any commercial or financial relationships that could be construed as a potential conflict of interest.
